# Matrix metalloproteinase-9, -10, and tissue inhibitor of matrix metalloproteinases-1 blood levels as biomarkers of severity and mortality in sepsis

**DOI:** 10.1186/cc8115

**Published:** 2009-10-02

**Authors:** Leonardo Lorente, María M Martín, Lorenzo Labarta, César Díaz, Jordi Solé-Violán, José Blanquer, Josune Orbe, José A Rodríguez, Alejandro Jiménez, Juan M Borreguero-León, Felipe Belmonte, Juan C Medina, Maria C LLimiñana, José M Ferrer-Agüero, José Ferreres, María L Mora, Santiago Lubillo, Manuel Sánchez, Ysamar Barrios, Antonio Sierra, José A Páramo

**Affiliations:** 1 Intensive Care Unit, Hospital Universitario de Canarias, Ofra, s/n. La Laguna, 38320, Santa Cruz de Tenerife, Spain; 2 Intensive Care Unit, Hospital Universitario Nuestra Señora de Candelaria, Crta del Rosario s/n. Santa Cruz de Tenerife, 38010, Spain; 3 Intensive Care Unit, Hospital San Jorge de Huesca, Avenida Martínez de Velasco no. 36, Huesca, 22004, Spain; 4 Intensive Care Unit, Hospital Insular, Plaza Dr. Pasteur s/n. Las Palmas de Gran Canaria, 35016, Spain; 5 Intensive Care Unit, Hospital Universitario Dr. Negrín, Barranco de la Ballena s/n. Las Palmas de Gran Canaria, 35010, Spain; 6 Intensive Care Unit, Hospital Clínico Universitario de Valencia, Avda. Blasco Ibáñez no. 17-19, Valencia, 46004, Spain; 7 Atherosclerosis Research Laboratory, CIMA-University of Navarra, Avda Pío XII no. 55, Pamplona, 31008, Spain; 8 Research Unit, Hospital Universitario de Canarias, Ofra, s/n. La Laguna, 38320, Santa Cruz de Tenerife, Spain; 9 Laboratory Deparment, Hospital Universitario de Canarias, Ofra, s/n. La Laguna, 38320, Santa Cruz de Tenerife, Spain; 10 Laboratory Department, Hospital San Jorge de Huesca, Avenida Martínez de Velasco no. 36, Huesca, 22004, Spain; 11 Microbiology Department, Hospital Universitario de Canarias, Ofra, s/n. La Laguna, 38320, Santa Cruz de Tenerife, Spain

## Abstract

**Introduction:**

Matrix metalloproteinases (MMPs) play a role in infectious diseases through extracellular matrix (ECM) degradation, which favors the migration of immune cells from the bloodstream to sites of inflammation. Although higher levels of MMP-9 and tissue inhibitor of matrix metalloproteinases-1 (TIMP-1) have been found in small series of patients with sepsis, MMP-10 levels have not been studied in this setting. The objective of this study was to determine the predictive value of MMP-9, MMP-10, and TIMP-1 on clinical severity and mortality in a large series of patients with severe sepsis.

**Methods:**

This was a multicenter, observational, and prospective study carried out in six Spanish Intensive Care Units. We included 192 (125 surviving and 67 nonsurviving) patients with severe sepsis and 50 age- and sex-matched healthy controls in the study. Serum levels of MMP-9, MMP-10, TIMP-1, tumor necrosis factor (TNF)-alpha, and interleukin (IL)-10 were measured in patients with severe sepsis at the time of diagnosis and in healthy controls.

**Results:**

Sepsis patients had higher levels of MMP-10 and TIMP-1, higher MMP-10/TIMP-1 ratios, and lower MMP-9/TIMP-1 ratios than did healthy controls (*P *< 0.001). An association was found between MMP-9, MMP-10, TIMP-1, and MMP-9/TIMP-1 ratios and parameters of sepsis severity, assessed by the SOFA score, the APACHE-II score, lactic acid, platelet count, and markers of coagulopathy. Nonsurviving sepsis patients had lower levels of MMP-9 (*P *= 0.037), higher levels of TIMP-1 (*P *< 0.001), lower MMP-9/TIMP-1 ratio (*P *= 0.003), higher levels of IL-10 (*P *< 0.001), and lower TNF-α/IL-10 ratio than did surviving patients. An association was found between MMP-9, MMP-10, and TIMP-1 levels, and TNF-α and IL-10 levels. The risk of death in sepsis patients with TIMP-1 values greater than 531 ng/ml was 80% higher than that in patients with lower values (RR = 1.80; 95% CI = 1.13 to 2.87;*P *= 0.01; sensitivity = 0.73; specificity = 0.45).

**Conclusions:**

The novel findings of our study on patients with severe sepsis (to our knowledge, the largest series reporting data about MMP levels in sepsis) are that reduced MMP-9/TIMP-1 ratios and increased MMP-10 levels may be of great pathophysiologic significance in terms of severity and mortality, and that TIMP-1 levels may represent a biomarker to predict the clinical outcome of patients with sepsis.

## Introduction

Matrix metalloproteinases (MMPs) are a family of zinc-containing endoproteinases implicated in degradation and remodelling of the extracellular matrix (ECM). They can be classified broadly by substrate specificity into collagenases (MMP-1, -8, and -13), gelatinases (MMP-2 and -9), stromelysins (MMP-3, -10, -11), elastases (MMP-7 and -12), and membrane-type (MT-MMPs, MMP-14, -15, -16, and -17). MMPs have a role in normal physiologic functions such as the menstrual cycle, morphogenesis, tissue remodelling and angiogenesis, and in diseases with abnormal ECM turnover, such as arthritis, tumor invasion, aneurysm formation, and atherosclerosis [1,2]. Regulation of MMP activity is carried out by specific tissue inhibitors of matrix metalloproteinases (TIMPs) [1,2].

MMPs play a role in infectious diseases when the host immune system is challenged by an invading organism, facilitating the recruitment of leukocytes from the bloodstream; these migrate to the site of infection for eradication of the pathogen (by proteolysis of the basement membrane) and for modulating the inflammatory response [3]. The action of MMPs and TIMPs has been reported in the coagulation/fibrinolytic system [4-6]; thus the MMP/TIMP system may play a role in the coagulation/fibrinolytic response to sepsis.

Small clinical studies (with fewer than 40 patients) have shown higher plasma levels of MMP-9 [7-13] and TIMP-1 [9,11,13] in sepsis patients as compared with those observed in controls, and higher levels of TIMP-1 [11] or MMP-9 [12] in nonsurviving than in surviving patients. However, no correlation between MMP levels and different indicators of severity in sepsis were reported, except for MMP-9 and Acute Physiology and Chronic Health Evaluation (APACHE)-II score [12]. It was recently suggested that MMP-10 plays a role in the development of atherosclerosis [14-16], and *in vitro *studies found increased MMP-10 levels after infective stimulation of human [17] and mice [18] airway epithelial cells; however, no studies assessing MMP-10 levels have been reported in sepsis.

Thus, the objective of this study was to determine the influence of the circulating levels of MMP-9, MMP-10, and TIMP-1 on the severity and mortality of patients with sepsis in a large cohort.

## Materials and methods

### Design and subjects

A multicenter, observational, prospective study was carried out in six Spanish Intensive Care Units. The study was approved by the Institutional Review Boards of the six hospitals, and informed consent from the patients or from the family members was obtained. In total, 192 patients with severe sepsis (mean age, 58 years; 66% men) and 50 age- and sex-matched healthy controls (mean age, 55 years; 73% men) were included.

The diagnosis of sepsis and severe sepsis was established according to the International Sepsis Definitions Conference [19]. Sepsis was defined as a documented or suspected infection (defined as a pathologic process induced by a microorganism) and some of the following parameters:

#### One

General parameters: fever (core temperature higher than 38.3°C), hypothermia (core temperature lower than 36.0°C), tachycardia (heart rate greater than 90 beats/min), tachypnea (respiratory rate higher than 30 breaths/min), altered mental status, significant edema or positive fluid balance (higher than 20 ml/kg over a 24-hour period), hyperglycemia (plasma glucose higher than 110 mg/dl) in the absence of diabetes.

#### Two

Inflammatory parameters: leukocytosis (white blood cell count higher than 12,000/mm^3^), leukopenia (white blood cell count lower than 4,000 mm^3^), normal white blood cell count with a percentage of immature forms higher than 10%, plasma C-reactive protein more than 2 standard deviations above the normal value, plasma procalcitonina more than 2 standard deviations above the normal value.

#### Three

Hemodynamic parameters: arterial hypotension (systolic blood pressure lower than 90 mm Hg, mean arterial blood pressure lower than 70 mm Hg, or decrease of systolic blood pressure from the baseline to higher than 40 mm Hg), mixed venous oxygen saturation higher than 70%, cardiac index higher than 3.5 l/min/m^2^.

#### Four

Organ dysfunction: arterial hypoxemia (pressure of arterial oxygen/fraction inspired oxygen (PaO_2_/FIO_2_) ratio <300), acute oliguria (urine output less than 0.5 ml/kg/h for at least 2 hours), creatinine increase of 0.5 mg/dl or more, coagulation abnormalities defined as international normalized ratio (INR) more than 1.5 or activated partial thromboplastin time (aPTT) more than 60 seconds, ileus (absent bowel sounds), thrombocytopenia (platelet count less than 100,000/μl), hyperbilirubinemia (plasma total bilirubin more than 4 mg/dl).

#### Five

Tissue perfusion parameters: hyperlactatemia (more than 3 mmol/l), decreased capillary refill or mottling.

Severe sepsis was defined as sepsis complicated by organ dysfunction.

Exclusion criteria were age younger than 18 years, pregnancy, lactation, human immunodeficiency virus (HIV), white blood cell count less than 1,000/μl, solid or hematologic tumor, or immunosuppressive, steroid, or radiation therapy.

### Variables recorded

The following variables were recorded for each patient: sex, age, diabetes mellitus, chronic obstructive pulmonary disease (COPD), site of infection, creatinine, leukocytes, lactic acid, platelets, INR, aPTT, and the Acute Physiology and Chronic Health Evaluation II (APACHE II) score [20], Sepsis-related Organ Failure Assessment [SOFA] score [21], and ICU mortality (defined as the death of the patient in the ICU).

Blood samples were collected from 192 patients with severe sepsis at the time of the diagnosis (within the first 2 hours after the diagnosis of severe sepsis) and from 50 age- and sex-matched controls.

### MMP-9, MMP-10, TIMP-1, TNF-α, and IL-10 assays

Serum separator tubes (SSTs) were used to determine MMPs and TIMP-1 concentration in serum. Venous blood samples were taken and centrifuged within 30 minutes at 1,000 *g *for 15 minutes, and the serum was removed and frozen at -80°C until measurement. MMP-9, MMP-10, and TIMP-1 were assayed with specific ELISA (Quantikine, R&D Systems, Abingdon, UK) according to the manufacturer's instructions with a serum dilution of 1:80, 1:2, and 1:100, respectively. The interassay coefficients of variation (CV) were less than 8% (n = 20), and the detection limits for the assays were 0.31 ng/ml, 78.1 pg/ml, and 0.15 ng/ml. TNF-α and IL-10 serum levels were measured with a solid-phase, chemiluminescence immunometrics assays kit (Immulite, Siemens Healthcare Diagnostics Products, Llanberis, UK); and the interassay coefficients of variation (CVs) were less than 6.5% (n = 20) and less than 9.9% (n = 40), and the detection limits for the assays were 1.7 pg/ml and 1 pg/ml, respectively.

### Statistical methods

Continuous variables are reported as medians and interquartile ranges. Categoric variables are reported as frequencies and percentages. Comparisons of continuous variables between groups were carried out by using the Wilcoxon-Mann-Whitney test. Comparisons between groups on categoric variables were carried out with the χ^2 ^(chi-square) test. The association between continuous variables was carried out by using the Spearman rank correlation coefficient or the Spearman rho coefficient. Receiver operation characteristic (ROC) curves were constructed to represent the goodness-of-fit of TIMP-1, lactic acid, and SOFA scores as criterion variables and mortality as the response variable. Relative risk and 95% confidence intervals were calculated as measurements of the clinical impact of the predictor variables. A *P *value of less than 0.05 was considered statistically significant. Statistical analyses were performed with SPSS 17.0 (SPSS Inc., Chicago, IL, USA) and NCSS 2000 (Kaysville, Utah, USA).

## Results

Baseline clinical characteristics and the median values (25^th ^to 75^th ^percentiles) of MMP-9, MMP-10, and TIMP-1 in sepsis patients and controls are shown in Table 1. No significant differences were found between 192 sepsis patients and 50 controls in terms of age and sex. Higher serum levels of MMP-10 (*P *< 0.001) and TIMP-1 (*P *< 0.001), and nonsignificantly higher levels of MMP-9 were observed in the group of patients compared with controls. The MMP-9/TIMP-1 ratio was markedly reduced in patients (*P *< 0.001), whereas the MMP-10/TIMP-1 ratio was significantly increased (*P *< 0.001).

**Table 1:** Comparison of MMP-9, MMP-10, and TIMP-1 serum levels between sepsis patients and controls (median and 25^th ^to 75^th ^percentiles are shown)

	Controls (n = 50)	Sepsis patients (n = 192)	*P*
Gender female: number (%)	13 (26.0)	64 (33.3)	0.11
Age (years)	57 (50-63)	60 (49-70)	0.39
MMP-9 (ng/ml)	498 (350-735)	676 (308-1,164)	0.07
TIMP-1 (ng/ml)	226 (213-241)	618 (445-831)	<0.001
MMP-10 (pg/ml)	466 (288-614)	1,880 (1,217-3,285)	<0.001
MMP-9/TIMP-1 (ratio)	2.19 (1.57-3.01)	1.16 (0.49-2.24)	<0.001
MMP-10/TIMP-1 (ratio)	2.07 (1.17-2.84)	3.09 (2.08-5.06)	<0.001

MMP = Matrix metalloproteinase; TIMP = tissue inhibitor of matrix metalloproteinase.

Comparisons of demographic and clinical parameters between nonsurviving (n = 67) and surviving sepsis patients (n = 125) are shown in Table 2. Whereas no differences were observed regarding age, sex, COPD, site of infection, and leukocytes; the nonsurviving sepsis patients showed a higher incidence of diabetes mellitus, higher levels of lactic acid and creatinine, prolonged aPTT, and reduced platelet count, together with increased SOFA and APACHE-II scores. Moreover, higher levels of TIMP-1 (*P *< 0.001), reduced MMP-9 (*P *= 0.037), and a nonsignificant increase of MMP-10 were found in nonsurviving as compared with surviving sepsis patients (Table 3). The ratio between MMP-9 and TIMP-1 was decreased in nonsurviving patients, whereas no differences in the MMP-10/TIMP-1 ratio were found. Finally, no significant differences in the levels of MMPs and TIMP-1 in relation to the presence of diabetes were found.

**Table 2:** Demographic and clinical parameters of surviving and nonsurviving sepsis patients (median and 25^th ^to 75^th ^percentiles or percentage when indicated are shown)

	Survivors (n = 125)	Nonsurvivors (n = 67)	*P*
Gender female: number (%)	40 (31.2)	24 (37.5)	0.27
Age: median years (percentile 25-75)	56 (47-69)	62 (52-71)	0.15
Diabetes mellitas: number (%)	25 (19.8)	24 (37.5)	0.02
COPD: number (%)	17 (13.5)	10 (15.6)	0.67
Site of infection			0.82
• Respiratory: number (%)	67 (53.2)	38 (59.4)	
• Abdominal: number (%)	28 (22.2)	13 (20.3)	
• Other sites: number (%)	31(24.6)	13 (20.3)	
APACHE-II score: median (percentile 25-75)	19 (14-22)	24 (18-29)	<0.001
Creatinine (mg/dl): median (percentile 25-75)	1.2 (0.80-2.05)	1.6 (0.9-2.8)	0.02
Leukocytes: median/mm^3 ^(percentile 25-75)	14,600 (8,900-20,050)	15,200 (9,050-20,625)	0.39
Lactic acid: median mmol/L (percentile 25-75)	2,00 (1.20-3.70)	3.95 (1.47-6.55)	<0.001
Platelets: median/mm^3 ^(percentile 25-75)	210,000 (127,000-273,000)	139,000 (63,000-218,250)	<0.001
INR: median (percentile 25-75)	1.27 (1.10-1.50)	1.42 (1.10-1.66)	0.17
aPTT: median seconds (percentile 25-75)	30 (26-39)	39 (30-47)	<0.001
SOFA store: median (percentile 25-75)	9 (7-11)	12 (9-14)	<0.001
• Respiratory: median (percentile 25-75)	3 (2-3)	3 (2-3)	0.07
• Hematologic: median (percentile 25-75)	0 (0-1)	1 (0-2)	<0.001
• Hepatic: median (percentile 25-75)	0 (0-1)	0 (0-1)	0.75
• Cardiovascular: median (percentile 25-75)	4 (4-4)	4 (4-4)	0.006
• Neurologic: median (percentile 25-75)	0 (0-1)	0 (0-3)	0.22
• Renal: median (percentile 25-75)	0 (0-2)	2 (0-4)	<0.001

APACHE II = Acute Physiology and Chronic Health Evaluation; aPTT = activated partial thromboplastin time; COPD = chronic obstructive pulmonary disease; INR = international normalized ratio; SOFA = Sepsis-related Organ-failure Assessment score.

**Table 3:** Comparison of MMP-9, MMP-10, TIMP-1, TNF-α, and IL-10 serum levels between surviving and nonsurviving sepsis patients (median and 25^th ^to 75^th ^percentiles are shown)

	Survivors (n = 125)	Nonsurvivors (n = 67)	*P*
MMP-9: median ng/ml (percentile 25-75)	784 (371-1222)	554 (240-1044)	0.037
TIMP-1: median ng/ml (percentile 25-75)	573 (422-724)	797 (499-1,012)	<0.001
MMP-10: median pg/ml (percentile 25-75)	1,850 (1,187-2,956)	2,284 (1,262-4,329)	0.09
MMP-9/TIMP-1 ratio: median (percentile 25-75)	1.39 (0.63-2.42)	0.82 (0.28-1.66)	0.003
MMP-10/TIMP-1 ratio: median (percentile 25-75)	3.12 (2.14-5.06)	2.97 (1.72-5.21)	0.46
TNF-α: median pg/ml (percentile 25-75)	30 (19-51)	34 (18-70)	0.38
IL-10: median pg/ml (percentile 25-75)	10 (5-37)	36 (9-103)	<0.001
TNF-α/IL-10 ratio: median (percentile 25-75)	2.49 (1.39-3.92)	1.20 (0.47-2.38)	<0.001

IL = interleukin; MMP = matrix metalloproteinase; TIMP = tissue inhibitor of matrix metalloproteinase; TNF = tumor necrosis factor.

Correlations between MMPs, TIMP-1, and severity of sepsis parameters are shown in Table 4. MMP-9 negatively correlated with SOFA, lactic acid, and coagulopathy markers (all *P *< 0.001) and positively with platelet count (*P *< 0.001). In contrast, TIMP-1 positively correlated with SOFA, lactic acid, and markers of coagulopathy (all p < 0.001). MMP-10 also correlated positively with SOFA and lactic acid (*P *< 0.001) and negatively with platelets (*P *< 0.001). Interestingly, although the MMP-9/TIMP-1 ratio showed significant correlations with all parameters of severity, no differences were found for the MMP-10/TIMP-1 ratio.

**Table 4:** Correlation between MMP-9, MMP-10, and TIMP-1 serum levels with lactic acid, SOFA, platelets, and coagulation markers in sepsis patients

	Lactic acid (mmol/L)	APACHE-II (points)	SOFA (points)	Platelet count (platelets/mm^3^)	aPTT (seconds)	INR (ratio)
MMP-9: ng/ml	Rho = -0.31	Rho = -0.34	Rho = -0.37	Rho = 0.48	Rho = -0.28	Rho = -0.28
	P < 0.001	P < 0.001	P < 0.001	P < 0.001	P = 0.001	P = 0.001
TIMP-1: ng/ml	Rho = 0.51	Rho = 0.38	Rho = 0.42	Rho = -0.24	Rho = 0.29	Rho = 0.41
	P < 0.001	P < 0.001	P < 0.001	P = 0.001	P < 0.001	P < 0.001
MMP-10: pg/ml	Rho = 0.29	Rho = 0.33	Rho = 0.36	Rho = -0.24	Rho = 0.13	Rho = 0.22
	P < 0.001	P < 0.001	P < 0.001	P < 0.001	P = 0.13	P = 0.008
MMP-9/TIMP-1 ratio	Rho = -0.45	Rho = -0.42	Rho = -0.48	Rho = 0.49	Rho = -0.38	Rho = -0.4
	P < 0.001	P < 0.001	P < 0.001	P < 0.001	P < 0.001	P < 0.001
MMP-10/TIMP-1 ratio	Rho = 0.01	Rho = 0.11	Rho = 0.04	Rho = -0.08	Rho = -0.09	Rho = -0.03
	P = 0.95	P = 0.13	P = 0.77	P = 0.30	P = 0.29	P = 0.72

aPTT = Activated partial thromboplastin time; INR = international normalized ratio; MMP = matrix metalloproteinase; rho = Spearman's rank correlation coefficient; SOFA = Sepsis-related Organ-Failure Assessment score; TIMP = tissue inhibitor of matrix metalloproteinase.

Inflammatory status was assessed in sepsis patients by measuring TNF-α and IL-10, to elucidate whether it could account for differences observed in MMPs and TIMP-1. Nonsurviving sepsis patients exhibited much higher levels of IL-10 than did the survivors, whereas no differences could be observed in TNF-α (Table 3). Moreover, IL-10 positively correlated with TIMP-1 and MMP-10, whereas a negative association could be observed for MMP-9 (Table 5).

**Table 5:** Correlation between MMP-9, MMP-10, and TIMP-1 with TNF-α and IL-10 serum levels

	TNF-α (pg/ml)	IL-10 (pg/ml)	TNF-α/IL-10 ratio
MMP-9: ng/ml	rho = -0.25	rho = -0.38	rho = 0.33
	p = 0.001	p < 0.001	p < 0.001
TIMP-1: ng/ml	rho = 0.56	rho = 0.50	rho = -0.26
	p < 0.001	p < 0.001	p = 0.001
MMP-10: pg/ml	rho = 0.35	rho = 0.30	rho = -0.16
	p < 0.001	p < 0.001	p = 0.04

SOFA = Sepsis-related Organ Failure Assessment score; aPTT = Activated partial thromboplastin time; INR = International normalized ratio; MMP = Matrix metalloproteinase; TIMP = Iissue inhibitor of matrix metalloproteinase; IL = interleukin; rho = Spearman's rank correlation coefficient.

**Figure 1:**
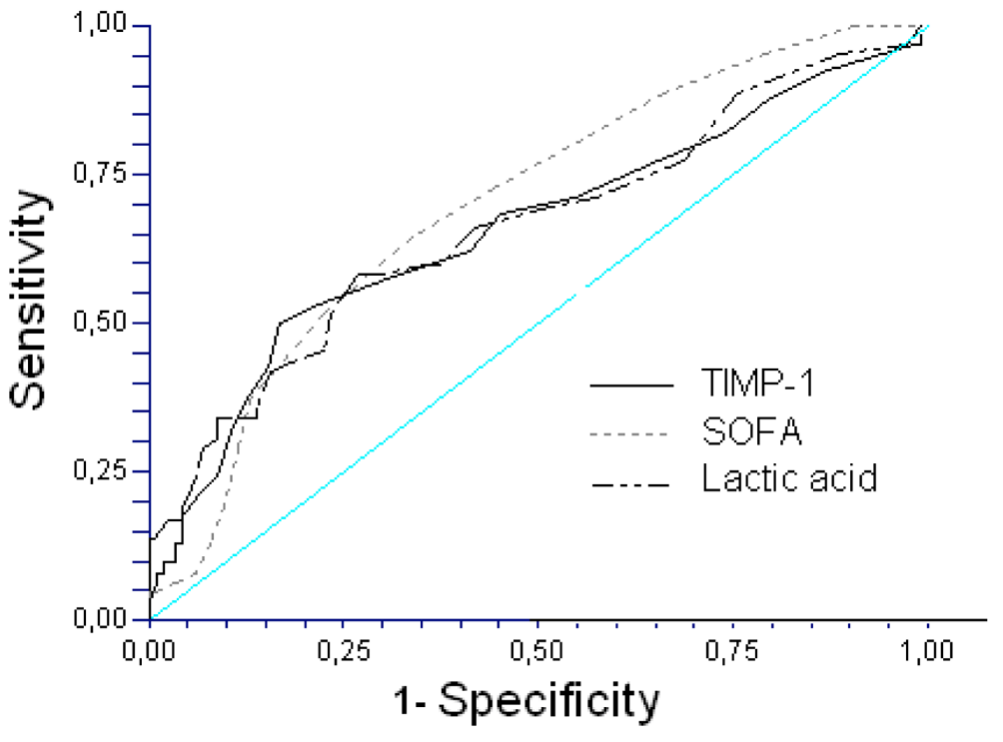
Receiver operation characteristic analysis using TIMP-1, lactic acid, and SOFA score as predictors of mortality.  The areas under the curves (AUC) for each predictor of mortality were the following: tissue inhibitor of matrix metalloproteinase (TIMP)-1 (AUC = 0.68; 95% CI = 0.59 to 0.76; *P *< 0.001), lactic acid (AUC = 0.67; 95% CI = 0.58 to 0.75; *P *< 0.001) and Sepsis-related Organ Failure Assessment score (SOFA) score (AUC = 0.71; 95% CI = 0.64 to 0.79; *P *< 0.001).

We performed an ROC analysis to determine whether the parameters analyzed could be used to predict outcomes in sepsis patients. Figure 1 shows the ROC analysis for mortality estimation. The areas under the curves as predictors of mortality were the following: TIMP-1 (AUC = 0.68; 95% CI = 0.59 to 0.76; *P *< 0.001), lactic acid (AUC = 0.67; 95% CI = 0.58 to 0.75; *P *< 0.001), and SOFA score (AUC = 0.71; 95% CI = 0.64 to 0.79; *P *< 0.001). The optimal cut-off for each predictor was TIMP-1 >531 ng/ml (RR = 1.80; 95% CI = 1.13 to 2.87;*P *= 0.01; sensitivity = 0.73; specificity = 0.45), lactic acid >3.1 mmol/L (RR = 2.13; 95% CI = 1.44 to 3.16;*P <*0.001; sensitivity = 0.55; specificity= 0.75), and SOFA score >8 points (RR = 3.12; 95% CI = 1.52 to 6.38;*P <*0.001; sensitivity = 0.82; specificity = 0.45).

## Discussion

To our knowledge, this study includes the largest series reporting data on MMP levels in sepsis. The most relevant findings were the following: (a) higher serum levels of MMP-10 and TIMP-1, and nonsignificantly higher MMP-9 levels in sepsis patients than in healthy controls; (b) a significant correlation between MMP-9, MMP-10, TIMP-1, and several indicators of severity in sepsis, including biomarkers of coagulation, lactic acid, APACHE-II, and SOFA scores; and (c) the nonsurviving sepsis patients had higher TIMP-1 levels, lower MMP-9/TIMP-1 ratios, and nonsignificantly higher MMP-10 levels than did surviving patients. Taken together, these results indicate that an alteration in the MMP-9/TIMP-1 ratio and MMP-10 levels may be of great pathophysiologic significance in sepsis patients.

Previous studies with small sample sizes (fewer than 40 patients) have shown higher levels of MMP-9 [7-13] and TIMP-1 [9,11,13] in sepsis patients than in controls. In our larger study, we found significantly higher levels of TIMP-1, reduced MMP-9/TIMP-1 ratios, and nonsignificantly higher MMP-9 levels in sepsis patients than in healthy controls. The small number of healthy controls may have contributed to the absence of significant differences in MMP-9 levels between the sepsis patients and these healthy controls. In addition, we report for the first time that sepsis patients have higher levels of MMP-10 than do controls.

Interestingly, we observed a significant correlation between MMP-10 and TIMP-1 and several markers of sepsis severity, such as SOFA and APACHE-II scores, lactic acid, and markers of coagulopathy; whereas MMP-9 negatively correlated with all the aforementioned parameters of sepsis severity. Therefore, besides the already known higher mortality rate in sepsis patients with increased lactic acid levels [22,23] and SOFA score [24], our results suggest that alterations in the MMP-9/TIMP-1 ratio and MMP-10 levels are associated with the severity of sepsis. However, we must note the apparent contradiction with a previous report of positive correlation between MMP-9 and APACHE-II score in sepsis patients [12].

After analyzing MMPs and TIMP-1 levels in relation to mortality, in our study, we found higher plasma levels of TIMP-1 and lower levels of MMP-9 in nonsurviving sepsis patients. Whereas higher levels of TIMP-1 were reported previously in nonsurviving patients [11], conflicting results regard MMP-9 [11,12]. Nakamura [12] observed higher levels of MMP-9, whereas Hoffman [11] found no differences in MMP-9 in nonsurviving sepsis patients. The reduced size of previous studies, particularly the group of nonsurvivors, could be affecting their statistical power and thus account for the apparent contradictory results. Although MMP-9 is secreted mainly by leukocytes [3], the observed differences cannot be explained by the leukocyte numbers, which were similar in both nonsurviving and surviving patients. Because TNF-α and IL-10 have been shown to modulate MMP-9 and TIMP-1 expression, we explored circulating levels of these cytokines. Although similar TNF-α levels were found in both groups, the augmented IL-10 observed in nonsurvivors could be responsible for reduced MMP-9 and increased TIMP-1 found in nonsurviving sepsis patients, because this anti-inflammatory cytokine has been shown to induce TIMP-1 and reduce MMP-9 expression in endothelium/monocyte cocultures [25].

When we performed ROC curve analysis to represent the goodness-of-fit of studied variables for predicting mortality, we found that TIMP-1 was a good predictor of mortality, compared with two well-established indicators for the same outcome: lactic acid levels and SOFA score. This result confirms previous observations from Hoffman *et al*. [11], showing that TIMP-1 and APACHE-II were predictors for outcome in 37 patients and reporting a relative risk of 4.5 for the cut-off point of TIMP-1 chosen, but with a large confidence interval (1.14 to 17.6). One strength of the present study is the large sample size that allowed us to increase the accuracy of the estimated parameters. In our study of 192 patients, the cut-off point presented a narrower confidence interval (relative risk, 1.8; 95% CI, 1.13 to 2.87). The TIMP-1 levels found in our study are lower, as described in previous studies, probably because of the use of different commercial kits in the TIMP-1 assay. According to the package insert of the kit that we used, mean TIMP-1 serum levels drawn from 60 apparently healthy volunteers were 190 ng/ml. In our study, median TIMP-1 serum levels in healthy controls were 226 ng/ml. In the study by Hoffmann *et al*. [11], the mean plasma levels of TIMP-1 in 37 healthy controls were 742 ± 34 ng/ml by using other commercial ELISA kits to determine TIMP-1 in plasma (Biotrak; Amersham Biosciences, Freiburg, Germany). Another potential explanation could be the existence of differences in the patient characteristics of each series; however, the APACHE-II score was not different from that in the previous study published by Hoffmann *et al*. In our study, the median APACHE-II scores were 19 and 24 in surviving and nonsurviving patients, respectively; and in the study by Hoffmann *et al*. [11], the mean APACHE-II scores were 14 and 23 in surviving and nonsurviving patients, respectively.

The role of MMPs/TIMPs in sepsis remains unclear; but the results of some studies indicate that MMPs play a certain role in the recruitment of leukocytes from the bloodstream to the site of infection [26-28], and in the inflammation [29-37] and coagulation/fibrinolysis response [38-41]. The migration of immune cells from the bloodstream to sites of inflammation requires MMP-mediated proteolysis of the basement membrane, as reported *i*n *vitro *[26] and in animal models [27,28]. MMPs may play a role in the inflammatory process because they modulate [29-32] and are modulated by cytokines [33-37]. MMPs have been found to promote the release of tumor necrosis factor (TNF)-α [29], to activate pro-interleukin (pro-IL)-1β [30], to cleave the activated form of IL-1β [31], and to convert IL-8 into a fragment 10 times more active than the parent molecule [32]. MMPs are secreted in response to cytokines such as TNF-α [33] and IL-1β [34] and are downregulated by diverse cytokines including interferon (IFN)-γ [35], IL-4 [36], and IL-10 [37]. Steroids, progesterone, and retinoids also downregulate MMPs [42]. Animal models have shown that endotoxinemia leads to the release of MMP-9 and endotoxin-induced shock in mice and that MMP-9-deficient mice were resistant to endotoxin-induced shock [43]. The relation between coagulation and inflammation in sepsis is already known [44-46]; and it is possible that MMPs/TIMPs may also play a role in the coagulation/fibrinolysis response in sepsis, as suggested by studies showing that MMP-9 inhibits platelet aggregation [39,40] and a positive correlation between TIMP-1 and PAI-1 [38].

All this indicates that sepsis is a complex clinical process with an interconnection between inflammatory and coagulation response; the inflammatory mediators activate coagulation and, conversely, intravascular coagulation induces an inflammatory response. We believe that the lower MMP-9/TIMP-1 ratio and higher MMP-10 levels in nonsurvivors than in surviving patients found in our study may be associated with a higher inflammatory and prothrombotic/antifibrinolytic state, responsible for the capillary thrombosis, multiple organ dysfunction, and death.

From a therapeutic perspective, the development of modulators of MMP/TIMP activity could be used as a new class of drugs for the treatment of severe sepsis, as suggested by the beneficial effect of targeting MMPs with the administration of sub-inhibitory doses of tetracycline reported in animal models of sepsis [47,48].

Whereas the strength of our study was the relatively large sample size that allowed us to increase the accuracy of the analyzed parameters in relation to previous studies [11,12], some limitations should be recognized. No analysis of MMP-9, MMP-10, and TIMP-1 during follow-up was performed; thus, we were unable to establish the time course of MMP/TIMP activity in the surviving patients compared with the nonsurvivors; therefore, additional prospective studies are required. Measuring other inflammatory cytokines, such as IL-6, would be desirable to evaluate better the relation between MMP/TIMP activity and inflammatory response in this set of patients; however, the number of analytic determinations per patient in our study was limited by available economic support. Higher dispersion in variables measured in the sepsis group led us to increase its sample size, thus constraining the dimension of the control group within the available funding for this study. The relatively small sample size of the control group may have contributed to the absence of significant differences in MMP-9 levels between controls and sepsis patients. Including other control groups, such as critically ill but nonsepsis patients, would be desirable for future studies to elucidate whether observed changes are specific for the septic setting.

## Conclusions

The novel findings of our study on severe sepsis patients are that reduced MMP-9/TIMP-1 ratio and increased MMP-10 levels may be of great pathophysiologic significance in terms of severity and mortality; and that TIMP-1 levels may represent a biomarker to predict the clinical outcome of sepsis patients.

## Key messages

• MMP-9/TIMP-1 ratio and MMP-10 levels may be of great pathophysiologic significance in terms of severity and mortality in sepsis patients.

• Reduced MMP-9/TIMP-1 ratio and increased MMP-10 levels may represent new predictive biomarkers of severity in these patients.

• TIMP-1 levels may represent a biomarker to predict the clinical outcome of sepsis patients.

## Abbreviations

APACHE: Acute Physiology and Chronic Health Evaluation; ICU: Intensive Care Unit; MMP: matrix metalloproteinase; SOFA: Sepsis-related Organ-failure Assessment score; TIMP: tissue inhibitor of matrix metalloproteinase.

## Competing interests

The authors declare that they have no competing interests.

## Authors' contributions

LL was responsible for conceiving, designing, and coordinating the study, made substantial contributions to the acquisition of data analysis, and interpretation of data, and drafted the manuscript. MMM, LL, CD, JSV, JB, FB, JCM, MCL, JMFA, and JF made substantial contributions to the acquisition of data and provided useful suggestions. MLM, SL, MS, and AS made substantial contributions to the analysis and interpretation of data. JO and JAR carried out the determination of MPM-9 and TIMP-1 and made substantial contributions to the analysis and interpretation of data. JMBL and YB carried out the determination of TNF-α and IL-10 and made substantial contributions to the analysis and interpretation of data. AJ contributed to data analysis and manuscript review. JAP contributed to study design and made substantial contributions to the analysis and interpretation of data. All authors read and approved the manuscript.
